# Reflective practice in management of morbidly adherent placenta: A four year comparative retrospective study

**DOI:** 10.12669/pjms.42.5.13964

**Published:** 2026-05

**Authors:** Syeda Sitwat Faitma, Shandana Bawar, Wagma Haq, Saima Shahzadi Hussain

**Affiliations:** 1 Syeda Sitwat Faitma, FCPS, MBBS.Assistant Professor,Department of Obstetrics and Gynecology, MTI Lady Reading Hospital, Peshawar, Pakistan; 2 Shandana Bawar, FCPS, MRCOG, MBBS.Associate Professor, Department of Obstetrics and Gynecology,Prime Teaching Hospital, Peshawar Medical College, Peshawar, Pakistan; 3 Wagma Haq, FCPS, MBBS.Department of Obstetrics and Gynecology, SPR,MTI Hayatabad Medical Complex, Peshawar, Pakistan; 4 Shahzadi Saima Hussain, FCPS, MRCOG, MBBS.Associate Professor,Department of Obstetrics and Gynecology, MTI Lady Reading Hospital, Peshawar, Pakistan

**Keywords:** Morbidly adherent placenta, Placenta accreta spectrum, Placenta previa

## Abstract

**Objectives:**

To analyze trends in management of morbidly adherent placenta and their impact on pregnancy outcomes over a four-year period at a tertiary care hospital center.

**Methodology:**

This retrospective cross-sectional study, was conducted in obstetrics and gynecology unit, from September 2018 to August 2022. It included women with singleton pregnancies complicated by Morbidly adherent placenta (MAP), diagnosed either prenatally by ultrasound / Magnetic resonance imaging, or intraoperatively based on failure of placental separation following delivery after 28 weeks of gestation. Pregnancy outcomes, analyzed over four years and compared between two groups defined by time interval: A (2018 – 2020) and B (2020 – 2022) to assess evolving trends in the management and care of MAP over time.

**Results:**

A total of 47 women were included with MAP. Preterm delivery (<37 weeks) was more frequent 25(53.2%) in MAP. Uterine conservation was achieved in 27.7% of patients. Blood loss >1500 ml was observed in 85.1% of cases, 57.4% required ≥4 units of intraoperative red blood cell (RBC) transfusion. Postoperative ICU admission was more common with placenta percreta(PPC). A significant association for surgical techniques was seen between Group-A and B: p-value 0.02. Surgical techniques were less frequent in Group-A as compared to Group-B. (B = -1.08, p = 0.052).

**Conclusion:**

Morbidly adherent placenta (MAP) is frequently associated with type four placenta previa(PP) and preterm delivery. Cesarean hysterectomy remains the most frequently performed surgical intervention; uterine conservation was feasible in less invasive forms. Multidisciplinary approach and advanced surgical techniques, may contribute to less blood loss and the preservation of future fertility.

## INTRODUCTION

Morbidly adherent placenta (MAP) or placenta accreta spectrum(PAS) is an abnormal invasion of placenta in to the uterus and/or surrounding structures.^1^ It is graded into placenta accreta (PA), increta (PI) and percreta (PPC), depending upon the adherence or invasion of placenta to uterine decidua, myometrium, serosa or into surrounding adjacent organs,respectively.^1^ MAP may occur with or without placenta previa: defined as a placental edge within 20mm of internal OS in second trimester or persisting in the lower uterine segment in the third trimester.^2^ The incidence of MAP/PAS recently has risen from 1/533 to 1/272 hospital births.^3^ Globally increased prevalence of MAP is attributed to escalated cesarean section rates, however, upper segment MAP may be ascribed to previous history of manual removal of placenta, evacuations or endometritis.^4^

The MAP is associated with unpredictable and severe hemorrhage resulting in emergency surgery with need of blood transfusions, intensive care unit(ICU)/ high dependency unit(HDU) admissions and multi organ damage secondary to antepartum or postpartum hemorrhage.^5^ The planned early delivery in MAP, adds into prematurity and neonatal intensive care unit admissions (NICU).^6^ The difficulties in MAP management, are attributed to surgical procedure itself, distorted anatomy due to repeated cesarean sections or percreta, blood transfusions and its sequel, ICU/NICU care, as well as psychological issues.^7^ Vigilant antenatal, intrapartum and postpartum care with multidisciplinary input including obstetrician, anaesthetist, hematologist, blood bank, radiologist help in reducing morbidity and mortality associated with MAP. Focused history to determine risk factors for MAP, use of radiological investigations like ultrasound(US), Doppler and magnetic resonance imaging(MRI) techniques may add in formulating a safer management plan.^8^

Cesarean hysterectomy had long been the end treatment of MAP, compromising the fertility of a woman, but over the time other surgical options e.g. Uterovaginal balloon tamponade (UVBT), devascularization (DV), prophylactic Internal iliac balloon inflation(IIBI) etc. have been evolved to save future fertility.^9^ This study aimed to analyze trends in management of MAP and their impact on pregnancy outcomes over four years duration. This would facilitate the establishment of protocols and guidelines in the management of MAP, enhancing patient safety and alleviating burdens on healthcare professionals through a multidisciplinary management plan, thereby saving time, reducing surgery duration, and minimizing infant mortality.^10^

## METHODOLOGY

This retrospective cross-sectional study was conducted in the Department of Obstetrics and Gynecology at MTI, Lady Reading Hospital, Peshawar.

### Ethical Approval:

It was approved from the Institutional Review Board (IRB) REF: 430/LRH/MTI; dated July 18, 2022.

This was a four years study, from year September 2018 to August 2022. No prior sample size calculation was done because of retrospective study design. The four-year duration was chosen to ensure adequate case accumulation and to capture management variations within the same institutional protocols. Selection bias was minimized by selecting all the eligible record. Women with single pregnancy diagnosed with MAP, prenatal by ultrasound(US)/magnetic resonance imaging (MRI) or during surgery, by failure of placenta to deliver, following 28 weeks of gestation were included in study. While MAP with multiple pregnancy and complications like polyhydramnios, abruption, medical disorders and those who declined consent were excluded because of their confounding effects on preterm delivery, hemorrhage and pregnancy outcomes like surgical procedures, intensive care admissions. MAP was classified as placenta accreta (PA) when adherent to the decidua, and as placenta increta (PI) when penetrating the uterine muscle. Placenta percreta (PPC) involved invasion beyond the uterine muscles. Per operative, PA/ PI were diagnosed by allowing for spontaneous separation of placenta, followed by an attempt to remove by controlled cord traction. PPC was diagnosed on visualizing engorged placental vessels on the serosa or involving adjacent structures. Pregnancy outcomes were defined as,

1: Surgical procedures: (vaginal delivery(VD)/ cesarean section(CS)/ cesarean hysterectomy(CH)), 2: Additional surgical techniques to arrest bleeding: Uterovaginal packing with gauze(UVPG) / Balloon temponade (BT) /, Devascularization (DV)/ Internal iliac balloon inflation(IIBI) /Abdominal homeostatic packing with gauze(AHPG) /combine Devascularization and Uterovaginal packing with gauze(DVUVPG), 3: Per-operative blood loss (POBL), 4: Per-operative blood transfusion(POBT). 5: Transfer to Critical care unit(CCU) and 6:Multidisciplinary team involvement (MDT).

The pregnancy outcomes were analyzed over four years and clinical trends were compared between two Groups defined by time interval. Group-A constituted MAP cases between year September 2018 - August 2020 while Group-B included MAP cases between year September 2020 - August 2022.

### Statistical analysis:

Data was collected retrospectively, by reviewing hospital operative gynecology and labor registers, identifying cases with MAP and extract medical record and serial numbers. Patient charts and software were examined to collect demographic information and study variables while maintaining confidentiality Data was entered and analyzed on SPSS version 22. Mean and standard deviation was calculated for continuous variables while frequency and percentages were calculated for categorical variables. Data was presented in tables, graphs and pie charts. The comparison of two groups A and B were done. Chi-square test or Fischer exact test applied where applicable. P-value of <0.05 was considered significant. Regression analysis was made where applicable.

## RESULTS

A total of 47 patients were included in our study. PP was found in 45(95.7%) while 02 (4.2%) had normally situated placenta. Type four PP 29 (61%) was most frequently associated with MAP. MAP was classified as, PA 27 (57.4%), PI 14 (29.8%) and PPC 06 (12.8%). Primi gravida were 04 (8.5%), multi-gravida were 28(59.6%), grand multi-gravida were 10 (21.3%) and great grand multi-gravida were 05(10.6%). Preterm deliveries were 25(53.2%), while term deliveries were 22 (46.8%), p-value = 0.04. Table-I shows demographic characteristics of women with MAP.

**Table-I:** Demographic characteristics of women with morbidly adherent placenta.

	No.	Mean ± standard deviation	median
Maternal age	47	29.32 ± 7.5	31.00
Gravida	47	2.3404 ± .78786	2.0000
Para	47	2.0638 ± .94188	2.0000
Antenatal stay	47	13.4 ± 17.7	4.0
Postnatal stay	47	04± 0.9	4.0

**Fig.1:**
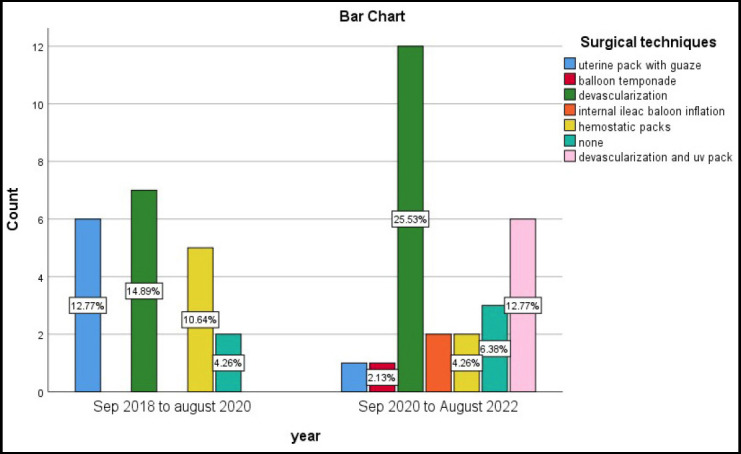
Distribution of surgical techniques to arrest bleeding in Group-A and Group-B.

Mode of delivery was predominantly CS 45(95.7%) while 02(4.3%) were VD. Among cesarean sections,34(75.6%) patients ultimately had CH. Surgical procedures in our study were classified as VD 02(4.3%), CS 11(24.4%) and CH (75.6%). Uterus was conserved in 13(27.7%). Additional surgical techniques to control hemorrhage during surgery is shown in Table-II. Blood loss of more than 1500ml was seen in 40(85.1%) while blood loss of lees than 1500ml was found in 07(14.9%) cases. Per-operative RBC transfusion of ≥ to 04 units was seen in 27(57.4%) while< 04 units was seen in 20(43.5%). Post operative management was done in HDU for 22(46.8%), 18(38.3%) were managed in ICU while 07(14.9%) patients were taken care in obstetrical ward. ICU care was seen more in PPC 04(66.7%) and PI 08(57.1%) compared with PA 06(22.2%). MDT included a single specialty at time of surgery was seen in 28(59.5%) patients. It included input of urologist 03(6.4%), Cardiovascular colleagues 04(8.5%) and interventional radiologist 03(6.3%). A multi-specialty/combine multidisciplinary team(CMDT) was seen in 19(40.4%) patients with MAP. Physiotherapist were involved in 18(38.3%) patients with MAP after surgery. Although overall management for MAP was more enhanced in Group-B, a non-significant association was seen between two groups when pregnancy outcomes were compared: (Frequency of MAP p-value 0.9, preterm delivery p-value 0.42,surgical procedures p-value 0.22, POBL p-value 0.10, RBC transfusion p-value 0.23,CCU management p-value 0.17 and MDT p=0.31).However, a significant association for additional surgical techniques to arrest bleeding was seen between Group-A and B: p-value 0.02. Devascularization and devascularization with UV pack were more common in Group-B whereas uterine pack with gauze was more prevalent in Group-A for less invasive MAP i.e. PA:Fig.1.

**Table-II:** Frequency of additional surgical techniques used to arrest bleeding.

Additional Surgical Technique	Number(%age)
None	05(10.6)
Devascularization	19(40.4)
Uterovaginal pack with gauze	07(14.9)
Abdominal homeostatic pack with gauze	07(14.9)
Combine devascularization with Uterovaginal pack with gauze	06(12.8)
Internal iliac balloon inflation	02(4.3)
Balloon tamponade	01(2.1)
Total	47(100)

Ordinal logistic regression with the logit link function was conducted to estimate the relationship between year of study and category of additional surgical techniques. The model showed that Group-A was a marginally non-significant predictor (Estimate = -1.082, p = 0.052). This implies that sophisticated surgical techniques were less frequent in earlier years (Group-A) and had a tendency to rise in Group-B. (B = -1.08, Wald = 3.77, p = 0.052). Table-III.

**Table-III:** Parameter estimates of ordinal logistic regression for additional surgical techniques.

Variable	Estimate (B)	Std. Error	Wald	p-value
Uterine pack with gauze	-2.263	0.515	19.34	<0.001
Balloon tamponade	-2.094	0.495	17.87	<0.001
Devascularization	-0.132	0.371	0.13	0.722
Internal iliac balloon inflation	0.042	0.370	0.01	0.910
Hemostatic packs	0.757	0.391	3.74	0.053
None	1.533	0.467	10.79	0.001
Group-A (Sep 2018 - Aug 2020)	-1.082	0.557	3.77	0.052
Group-B (Sep 2020 - Aug 2022): a	Reference	—	—	—

## DISCUSSION

Our study determined 47 MAP cases in four-years contrary to Srinivasan B et al, who identified 47 cases in 10 years, likely due to differences in study designs and settings.^9^ PP type four was frequently associated with MAP similar to Tehseen et al, who reported a high frequency of MAP with type four PP.^11^ PA was a common finding, similar to our study by Srinivasan et al.^9^ and Heena et al.^12^, the later substantiated her results with histopathological evidence. A regional retrospective study determined a hospital stay of 4-11 days with MAP and PP, almost similar to our study^13^.A local cross sectional study determined gestational age of more than 34 weeks for MAP at delivery,^14^ while a retrospective trial by Salmanian et al determined (28%) of PAS, delivered before 34 weeks.^15^ Frequency of preterm delivery was higher in our study, possibly because our operational definition was <37 weeks, compared with <34 weeks in the referenced study. Uterine conserving surgery were less compared to Srinivasan et al.^9^ and Aryananda et al.^16^ 33/43(76%) and 200/242(82%), respectively. Different selection criteria of participants, time of diagnosis of PAS and expertise of UAE and vascular surgeons availability at the time of surgery may explain the contrast in frequency of uterine conservation. Surgical techniques have evolved to manage postpartum hemorrhage and try saving uterus in MAP/PAS. Barinov SV et al.^17^ and Paping A et al.^18^ determined, DV, IIBI and BT, when used alone, did not reduce blood loss and mostly required CH. In contrast, Cho SB et al found IIBI to effectively reduce blood loss however, did not reduce hysterectomy rates.^19^

In our study, these techniques were employed in a recommended stepwise manner but could successfully conserved uterus in 13(27.7%) of cases. IIBI was a newly introduced technique in our setting and did not significantly reduce hemorrhage, possibly due to limited local experience compared to advance and routinely practice in referenced Korean study. Our research reported blood loss of more than 1500ml along with massive blood transfusion in PA. Salmanian B et al. reported no significant difference in blood loss with higher degree of placental invasion, despite frequent transfusion requirements.^20^ This may reflect greater vigilance and a lower threshold for hysterectomy in PI and PPC, while efforts to conserve uterus in PA often result in increased blood loss. We observed ICU care was frequent in severe placental invasion (PI and PPC) ending in CH, whereas PA was largely managed in HDU. Similarly, two regional studies have recommended ICU management for cases requiring hysterectomy.^9^,^13^ Greater surgical complexity, hemodynamical instability in case of PI, PPC increases ICU admissions. Similar to our study, several researches identified MDT care as a key determinant of patient safety and improved outcomes, thus ensuring quality care.^10^,^21^

We appraised the trends and practices of MAP management, in two Groups A and B. Study period Sept 2020-Aug 2022 (Group-B) utilized more complex and combined surgical techniques with clinical experience and multidisciplinary collaboration. Introduction of new surgical techniques (IIBI) was seen in Group-B with resultant decrease in conventional UVPG and uptake of modified BT, DV techniques. Though, the newly adopted techniques did not raise the number of uterine conserving surgeries in our study, similar findings were met in a case control study for IIBI.^22^ The planned surgeries in Group-B led to increase antenatal hospital stay and preparedness for postpartum hemorrhage algorithms, contributing to multi-modal surgical approaches in later years. Chandraharan et al. in their recent study emphasized the importance of referring MAP/ PAS, to centers with multi-specialty expertise, for better outcomes.^23^

### Strength of the study:

Strength of our study is the longitudinal assessment of a grave condition MAP over the four years thus closing the gap between audit and research. It identifies trends and evaluates its impact on pregnancy outcomes. The objective measurement of outcomes strengthens the validity of conclusions.

### Limitations:

However, the retrospective, single-center, cross-sectional design of this study limits the results which cannot be generalized. Potential selection bias and unmeasured confounding could not be excluded. Larger multi-center prospective studies are recommended to confirm these trends by evaluating the effectiveness of surgical techniques with clinical care.

## CONCLUSION

MAP is frequently associated with type four placenta previa(PP) and preterm delivery. Cesarean hysterectomy remains the most frequently performed surgical intervention; uterine conservation was feasible in less invasive forms. Multidisciplinary approach and advanced surgical techniques, may contribute to less blood loss and the preservation of future fertility.

### Recommendations:

Future studies on patient follow up and satisfaction rate after uterine conservation surgery, are needed to enhance and modify current MAP management.

### Authors’ Contribution:

**SSF and SB:** Conceived, designed, acquisition of data and interpretation of data, drafting and revising and final approval of the article. Responsible and accountable for the accuracy or integrity of the work. Helped in acquisition, interpretation and statistical analysis of data analysis, drafting and revising and final approval of the article. Responsible and accountable for the accuracy or integrity of the work. **WH and SSH:** Helped in reviewing, editing of manuscript, and revising and final approval of manuscript. Responsible and accountable for the accuracy or integrity of the work. All the authors have read and approved the final version.
